# What is Known About Muscle Strength Reference Values for Adults Measured by Hand-Held Dynamometry: A Scoping Review

**DOI:** 10.1016/j.arrct.2021.100172

**Published:** 2021-12-07

**Authors:** Marika Morin, Elise Duchesne, Jacinthe Bernier, Philippe Blanchette, Daphnée Langlois, Luc J. Hébert

**Affiliations:** a Department of Health Sciences and Centre intersectoriel en santé durable, Université du Québec à Chicoutimi (UQAC), Saguenay, Québec, Canada; b Interdisciplinary Research Group on Neuromuscular Disorders, Centre intégré universitaire de santé et de services sociaux du Saguenay-Lac-St-Jean, Jonquière, Canada; c Department of Rehabilitation, Université Laval, Quebec City, Québec Canada; d Center for Interdisciplinary Research in Rehabilitation and Social Integration, Centre intégré universitaire de santé et de services sociaux de la Capitale-Nationale, Quebec City, Québec, Canada; e Department of Radiology-Nuclear Medicine, Université Laval, Quebec City, Québec Canada

**Keywords:** Adult, Muscle strength, Reference values, Rehabilitation

## Abstract

**Objective:**

To map the body of existing literature regarding reference values of maximal isometric muscle strength (MIMS) of upper and lower limbs obtained with handheld dynamometers (HHD) in healthy adults to identify potential gaps in the literature and specify future research needs.

**Data Sources:**

A scoping review in which PubMed, EMBASE, CINAHL plus, PEDRO, and Cochrane databases were searched before May 1, 2020.

**Study Selection:**

All studies using standardized HHD protocols for the purpose of establishing reference values in healthy adult population were included. Two independent reviewers completed an initial screening of article titles and abstracts, and the remaining articles were read in their entirety and screened according to the inclusion and exclusion criteria. Disagreements were discussed, with recourse to a third reviewer when needed.

**Data Extraction:**

Data of the selected studies were extracted and charted by 2 independent reviewers using a tested data extraction grid to ensure method standardization. Data were subsequently merged to produce the complete final extracted data.

**Data Synthesis:**

Titles and abstracts of 4015 studies were screened, 46 articles were fully reviewed, and 9 manuscripts were selected for the final analysis. A large variability exists between protocols used for muscle testing in terms of type of device, measurement units, subject positioning, and muscle groups tested.

**Conclusion:**

The existing literature regarding HHD reference values of MIMS is scarce and presents gaps notably relating to strength units and well-described protocols with known psychometric properties, despite the development and increased availability of high quality HHD. This observation emphasizes the critical need to develop reference values in manual dynamometry in adults to optimize the use of manual dynamometry for diagnostic and prognostic decisions.

## Highlights

• Existing literature regarding handheld dynamometer (HHD) strength reference values is scarce.
• The current literature includes gaps relating to strength units used and well-described protocols.
• There is a critical need to develop HHD reference values in adults.
• Considering the increased availability of high quality HHD, this issue is urgent.

Muscle strength is a central component of function and movement. As such, it is essential to accomplishing daily living tasks and maintaining autonomy.^1^, ^2^, ^3^ Although muscle strength is known to be a good predictor of functional capacity among the general adult population, strength deficits are associated with physical limitations.^3^^,^^4^ For these reasons, evaluating this variable is a key component of physiotherapists' work; muscle strength reference values obtained from healthy adults allow clinicians to detect muscle weakness, quantify and identify the presence of neuromuscular impairment by comparing the values obtained to those of a healthy individual of the same age group and sex, to objectify patients' progress and to determine treatment effectiveness. 1, 2, 3, 4, 5

Many tools have been developed to obtain objective measurements of muscle strength, a component of muscle power, which is an important function of the neuromusculoskeletal system and movement according to the International Classification of Functioning, Disability and Health (B730 Muscle power functions ICF, https://apps.who.int/classifications/icfbrowser/). Manual muscle testing (MMT) is the most accessible and commonly used method. Although clinically feasible and quick to perform, this subjective method has poor psychometric properties and demonstrates significant limitations in detecting changes of strength over time.^6^, ^7^, ^8^, ^9^, ^10^ For example, Hébert et al^6^ showed that even when MMT is used by clinicians who have several years of experience and are using a more sensitive tool such as a 10-point scale, it cannot accurately classify patients and discriminate between patients with mild and severe impairments. Moreover, in patients with muscular dystrophy type 1 presenting with the late-onset phenotype, quantified muscle testing using a handheld dynamometer (HHD) revealed a strength loss of as much as 20.4%, whereas MMT testing suggested normal strength.^9^ At the other end of the spectrum, isokinetic dynamometry is a method with sound psychometric properties and is considered the criterion standard measurement of muscle strength. However, the equipment is costly and requires considerable space to accommodate, and extensive training of users is required.^11^ An interesting compromise between MMT and isokinetic dynamometry is quantified muscle testing using an HHD. The HHD is accessible, user-friendly, affordable, and has excellent psychometric properties, rendering it a top choice for the assessment of muscle strength impairments.^11^, ^12^, ^13^, ^14^, ^15^, ^16^ Maximal isometric muscle strength (MIMS) values obtained in some muscle groups with HHD are highly correlated with values obtained with isokinetic dynamometry, indicating good to excellent validity of both methods.^15^ However, it should be understood that the use of HHD is inevitably linked to different sources of error measurement depending on the muscle group assessed, the experience and training of the evaluators, and the standardization of the protocols.^17^, ^18^, ^19^ The most recent generation of HHD that can measure in both compression (push) and distraction (pull) modes, such as the Medup^a^ or the Chatillon,^b^ are frequently used in clinical settings.^20^ Make and break tests are commonly used to measure muscle strength with HHD. Performing a “make” test implies that the evaluator holds the HHD stationary, whereas the participant exerts a maximal force against it; for a “break” test, the evaluator has to exert enough force to break the isometric contraction produced by the person. In this study, we were only interested in “make” test protocols because “break” tests have questionable reliability according to our clinical experience and the literature, and this type of test exposes participants to a higher risk of injuries.^21^^,^^22^

Currently, to draw conclusions on the presence or absence of significant muscle impairments, MIMS values obtained from the affected muscle group are compared with those of the same muscle group on the contralateral side, assuming that the latter is healthy and experiences no neuromuscular impairment. However, this practice becomes problematic when individuals present bilateral strength deficits or when the supposed healthy side is not perfectly free of impairments. In these circumstances, the values obtained from the contralateral side cannot provide a valid comparator and, therefore, an “external” comparison according to muscle group may be necessary to identify muscle weakness. Moreover, even in the presumed absence of impairments, it remains difficult to determine if the muscle strength of the healthy side is appropriate and considered normal for a given individual of a given age and sex. Few studies have reported normative values of muscle strength in healthy populations for some muscle groups, making it difficult to address this important question. For example, Hogrel et al^23^ and Danneskiold‐Samsøe et al^24^ established normative strength values of several upper and lower limb muscle groups and the trunk with an isokinetic dynamometer and a force gauge fixed to an external structure. Unfortunately, as these devices are quite different from HHDs used by physiotherapists and are mainly used as research tools in conditions inaccessible to clinicians, these values cannot be used as a reference. Moreover, the protocols used in both studies differ from that developed with push-pull HHD, which considerably limits the clinical applicability of the reference values established by these authors. Hebert et al^20^ and Beenakker et al^25^ established reference values for several muscle groups of upper and lower limbs using push-pull HHD in the pediatric population, limiting the use of these values in individuals younger than 18 years old. It would therefore be relevant to know if similar clinically applicable data exist in the literature for adults. As a first-view approach to examine the research activity in this field, we conducted a scoping review avoiding the methodological shortcomings often found in rehabilitation scoping reviews.^26^

The main purpose of this scoping review was to map the existing literature regarding reference values of MIMS of upper and lower limb muscle groups obtained with HHD in healthy adults. The review will also serve to identify potential gaps in the literature and guide future research. Our principal hypothesis was that the current literature is incomplete, as it lacks reference values of MIMS for several muscle groups in adults using push-pull HHD.

## Methods

This scoping review was performed using the framework methodology presented in Khalil et al.'s *An Evidence-Based Approach to Scoping Reviews 2016*,^27^ which is based on the works of Arksey and O'Malley (2005),^28^ those of Levac, Colquhoun, and O'Brien (2010),^29^ as well as the Joanna Briggs Institute *Manual for Evidence Synthesis*.^30^ Our review complies with reporting guidance for the conduct of scoping reviews (ie, Preferred Reporting Items for Systematic Reviews and Meta-Analyses [PRISMA] extension for Scoping Reviews). In the literature on muscle strength assessment, the terms “reference values” and “normative values” are often considered synonymous. These values ​​are referred to as to the data set for muscle strength measurements, which are expected in a group of functional and healthy people. These values ​​allow comparisons to be made with measurements taken in the clinic so that the results obtained can be interpreted objectively. Therefore, to include all of the literature relevant to our scoping review, our research focused at both normative and reference values. However, for the purpose of this scoping review, the term reference values was defined as the value of a property obtained by observation or measurement on a reference individual and not in the context of randomized controlled trials or studies comparing healthy people to people with impairments and disabilities. In this scoping review, the studies considered were the ones using the following concept for reference values: isometric muscle strength reference values ​​correspond to quantifiable data of isometric muscle strength gathered from a large sample of the population representative of the general population. These values, ​measured several times in the same individual, must be obtained under carefully described conditions, allowing interpretation within the limits of their known metrological properties, and they represent what we would expect as muscle strength data in healthy adults.

### Research question

This scoping review aimed to improve our knowledge regarding the existence of reference values of quantified MIMS in healthy adults. The following questions were addressed in the review: (1) Is there a consensus and consistency in the use of the terms “reference values” vs “normative values”?, (2) What is known in quantified MIMS obtained with HHD in healthy adults?, and (3) Is there consensus concerning the protocols and methodology used for muscle testing with HHD to obtain reference values? These questions were built using the Population, Context, and Concept model in which healthy adults were the population, reference values of muscle strength were the concept, and the evaluation of muscle strength with HHD was the context.

### Data sources and searches

To identify the relevant literature, PubMed, EMBASE, CINAHL plus, PEDRO and Cochrane databases were searched. The search strings were “reference values/normative values,” “isometric muscle strength,” and “handheld dynamometry” (see supplemental fig 1, available online only at https://www.sciencedirect.com/journal/archives-of-rehabilitation-research-and-clinical-translation, for complete list of terms). After consulting and extracting articles from the databases, gray literature was searched in the RehabData and Proquest Dissertations databases, using the same search terms. The search strategy was reviewed and validated by a health sciences information specialist. After the initial search, duplicates were removed. The systematic literature search of databases was undertaken before January 13, 2020 and the search in the gray literature before May 1, 2020.

### Study selection

Two independent reviewers (D.L. and P.B.) completed an initial screening of article titles and abstracts according to the inclusion and exclusion criteria. The selected articles were kept for further analysis. To be included in the study, the articles had to concern testing protocol using HHD for the purpose of establishing reference values in healthy adult populations aged 18 years and older (ie, without any history of medical, neurological, and musculoskeletal impairments or any condition that could affect torque measurements), be written in French or English, and be available in full text. Studies addressing the following themes or populations were excluded: (1) animals, high level athletes, adults with pathologies or any other condition affecting muscle integrity; (2) measurements of spine force, nonisometric strength (isokinetic or isotonic methods) or hand grip strength; (3) studies where a “break test” approach was used; (4) case studies; (5) studies using a device other than an HHD; and (6) studies in which strength values of healthy participants were obtained in the context of randomized controlled trials or when comparing healthy individuals with those with impairments and disabilities. After the initial screening, the remaining articles were read in their entirety and screened twice by the same independent reviewers (DL and PB) to ensure their eligibility. Disagreements regarding eligibility were discussed by both reviewers and resolved by consensus, with recourse to a third reviewer (JB) when needed. References of selected articles were checked to identify other eligible articles not retained following the initial database search. Because scoping reviews do not entail the appraisal and exclusion of articles based on the quality of research methodology, no risk of bias assessment was undertaken.^27^

### Data extraction

Data of the selected studies were extracted and charted by 2 independent reviewers (M.M. and L.J.H.) using a data extraction grid to ensure method standardization (table 1). A beta version of the extraction grid was tested on 2 articles before the final grid was produced. The data from the extraction grids completed by the 2 independent reviewers were subsequently merged to produce the complete final extracted data.

**Table 1:** Data extraction grid.

Data Extracted
Authors
Year
Country
Study eligibility
Aims/objectives
Sample (type)
Level of activity
Age
Sex
Number of participants (total and per decade)
HHD model
Measurement units
HHD maximal capacity (N, lb, kg) Mode (compression/traction)
Contraction type
Instructions
Protocol reproducibility (positioning for measurement)
Muscle groups tested
Results
Limits reported
Other

### Data synthesis and analysis

The results were summarized in table format under 2 main themes: protocol variables and positioning descriptions for muscle testing. The protocol variables were subdivided into 5 items: HHD, units of measurement, testing procedure, muscle groups assessed, and positioning. The positioning item was subdivided in 5 categories: subject position (during the test), tested limb position, anatomic landmark for HHD placement, stabilization, and whether or not gravity was eliminated (limb placed in a neutral position in regard to gravity to eliminate the effect of segment weight) for each muscle group tested. Extracted data were analyzed, classified, and interpreted to map the breadth of the current existing knowledge regarding the research questions and to specify future research needs.

## Results

### Relevant literature identification

**Fig 1:**
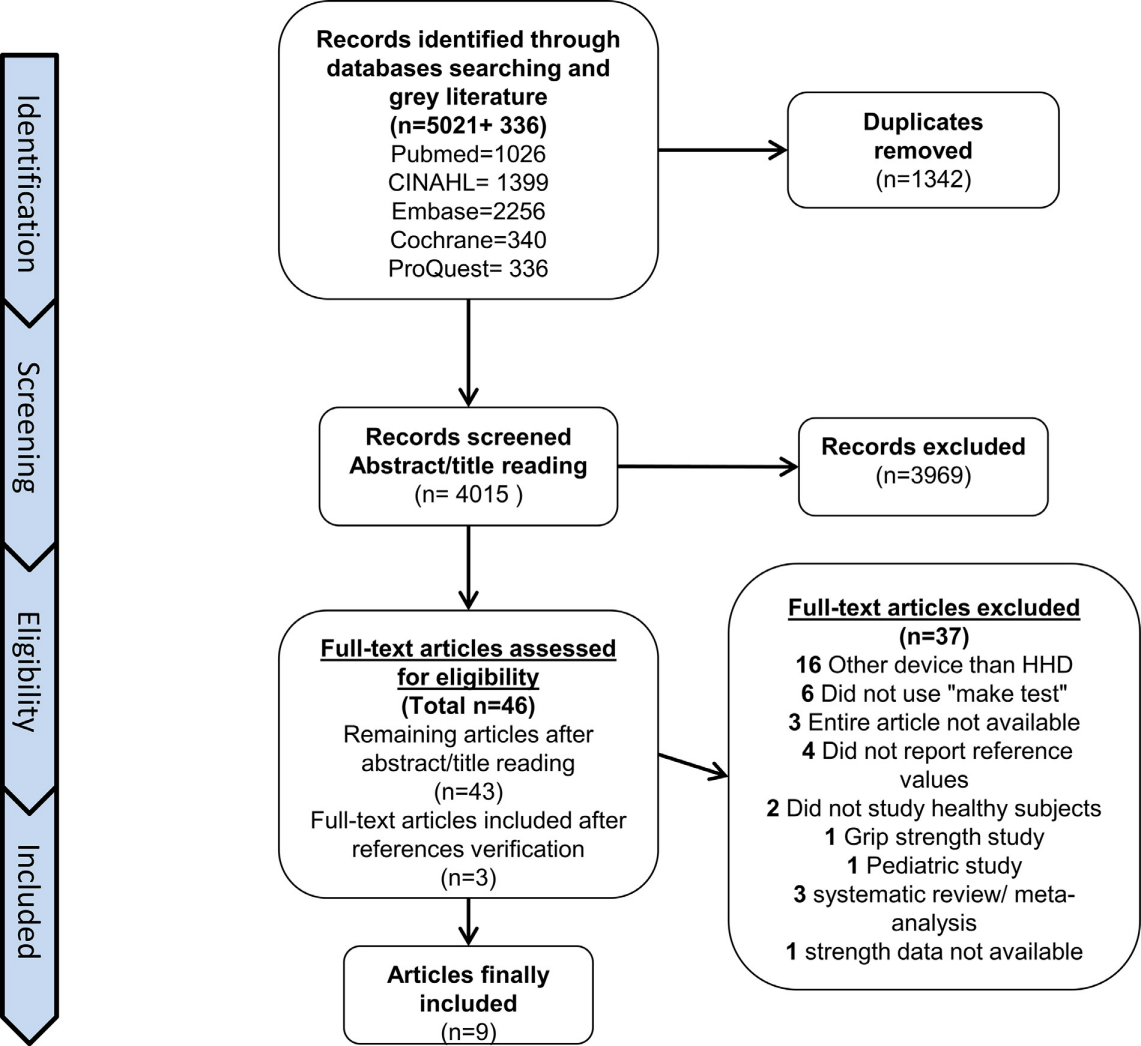
Flowchart of the systematic literature search according to the PRISMA statement.

As shown in figure 1, a total of 5021 studies were identified with the initial search in scientific literature databases and 336 papers were found in the gray literature by searching the Proquest Dissertations and Theses website. As 1342 duplicates were identified and excluded, 4015 studies were screened. Of these, 43 studies were selected based on titles and abstracts. Three articles were added after verification of references. During full-text screening of the remaining 46 articles, 35 papers were excluded by the 2 reviewers in accordance with the inclusion and exclusion criteria (see fig 1 for reasons for exclusion). Eleven articles were selected for the final data extraction. Two studies, Bohannon^31^ and Bohannon,^32^ were excluded, as they were a systematic review and a meta-analysis, respectively. These 2 studies included articles that were either already included in our scoping review or were excluded according to our eligibility criteria. Finally, the data from 9 articles were extracted, analyzed, and discussed.

### Study characteristics and data summary

Information regarding the selected studies is presented in table 2. The data regarding the protocol variables are summarized in table 3, and the data for the positioning for muscle testing are summarized in tables 4 (upper limb muscle groups) and 5 (lower limb muscle groups), respectively.

**Table 2:** Study characteristics.

**Authors**	**Year**	**Normative Values (N)/Reference Values (R)**	**Instruments and Measures**	**Testing Procedures**	**Muscle Groups Tested**	**Participants**	**Positioning and Protocol Reproducibility**
Al-Abdulwahab^39^	1999	ᴓ (preliminary baseline)	Model: Nicholas HHD Unit: N Maximal capacity: 1959 N (199.9 kg)	Contraction type: make test Mode: compression Gravity: neutralized (sitting position knee and hip flexed 90 degrees) Measures: 3 MIVC Contraction time: build force to a maximum over a 2-second period and maintain the MIVC for 5 seconds Rest time: 2 minutes Verbal stimuli: No Evaluator: 1 evaluator	LE: Knee: extension	Sample: convenience Ethnicity: Saudi Arabian Activity level: <2 hours\week Sex: male Age: 20-89 years Number: 160 Number per decade: 20s: 30 30s: 20 40s: 29 50s: 26 60s: 19 70s: 20 80s: 16	Reproducible Anatomical landmarks: yes Subject position: yes Joint/limb position: yes Stabilization: yes Pictures: no
Alvarenga et al^33^	2019	N	Model: MicroFET 2, Draper, USA Unit: kg Results expressed as %, normalized to bodyweight Maximal capacity: NS (1334 N)	Contraction type: make test Mode: compression Gravity: not specified Measures: 5 MIVC Contraction time: 5 seconds Rest time: 15 seconds Verbal stimuli: yes Evaluator: 2 evaluators Particularities: device fixed to the limb with a rigid belt secured to the wall with a suction cup	LE : Hip: Flexion/extension, RE/RI, ABD/ADD	Sample: convenience Ethnicity: NS Activity level: sedentary or sporadically active Sex: female Age: 20-29 years Number: 52 Number per decade: 52 (1 decade only)	Not reproducible Anatomical landmarks: yes Subject position: yes Joint/limb position: no Stabilization: No Pictures: no * reference to Thoborg et al.
Andrews et al^36^	1996	N and R	Model: Chatillon CSD400C Unit: lb Results expressed as %, normalized to bodyweight Maximal capacity: 512 N (115 lb) minutes of rest	Contraction type: make test Mode: compression Gravity: neutralized Measures: 2 MIVC Contraction time: 7 seconds (2 seconds progressive) Rest time: 1-2 minutes Verbal Stimuli: no Evaluator: 3 evaluators (with at least 8 years of experience) Particularities: Help for participant stabilization during knee flexion and extension	UE: Shoulder: flexion/extension, ABD and RE/RI Elbow: flexion/extension Wrist: extension LE: Hip: Flexion, ABD Knee: flexion/extension Ankle: dorsal flexion	Sample: convenience Ethnicity: NS Activity level: II on 4-point ordinal activity scale Sex: male and female Age: 50-79 years Number: 156 Number per decade: 50-59: 50 (25 men, 25 women) 60-69: 55 (26 men, 29 women) 70-79: 51 (26 men, 25 women)	Reproducible Anatomical landmarks: yes Subject position: yes Joint/limb position: yes Stabilization: yes Pictures: only 1 picture
Bohannon^40^	1986	ᴓ (preliminary information)	Model: HHD Spark instruments and Academics, Inc Unit: Lb and kg Maximal capacity: 60 lb (27.3 kg) (265 N)	Contraction type: make test Mode: compression Gravity: neutralized Measures: 1 contraction Contraction time: NS Rest time: NS Verbal stimuli: no Evaluator: 1 evaluator	UE: Shoulder: flexion/extension, ABD/ADD and RE/RI Elbow: flexion/extension Wrist: flexion/extension	Sample: NS Ethnicity: NS Activity level: 3 sedentary, 23 active, and 6 very active Sex: female Age: 20-40 years Number: 31 Number per decade: NA	Reproducible Anatomical landmarks: yes Subject position: yes Joint/limb position: yes Stabilization: yes Pictures: yes *Reference to another article Bohannon, 1986
Bohannon^41^	1996	ᴓ (data)	Model: Accuforce II Unit: N Results expressed in % of the mean actual force of participants 20-29 years Maximal capacity: 650 N	Contraction type: make test Mode: compression Gravity: neutralized Measures: 1 contraction Contraction time: build contraction 1-2 seconds and maximal contraction 4-5 seconds Rest time: NS Verbal stimuli: no Evaluator: 1 evaluator	UE: Shoulder: ABD Elbow: flexion Wrist: extension LE: hip: Flexion Knee: extension Ankle: dorsiflexion	Sample: NS Ethnicity: NS Activity level: NS Sex: female Age: 20-79 years Number: 123 Number per decade: >18 for each decade	Reproducible Anatomical landmarks: No Subject position: No Joint/limb position: No Stabilization: No Pictures: No *Reference to another article, Bohannon 1996
Bohannon et al^37^	1997	N and R	Model: Accuforce II (Amatek) Unit: N Results expressed in N and as %, normalized to bodyweight Maximal capacity: 650 N	Contraction type: make test Mode: compression Gravity: neutralized Measures: 2 MIVC Contraction time: build contraction 1-2 seconds and maximal contraction 4-5 seconds Rest time: 1-2 minutes Verbal stimuli: no Evaluator: One evaluator (more than 10 years of experience with HHD)	UE: Shoulder: extension, ABD and RE Elbow: flexion/extension Wrist: extension LE: Hip: Flexion, ABD *Knee:* extension *Ankle:* dorsiflexion	Sample: convenience Ethnicity: NS Activity level: 2 on ordinal scale of Saltin and Grimby Sex: male and female Age: 20-79 years Number: 231 (106 men, 125 women) Number per decade: 20-29: 38 (16 men, 22 women) 30-39: 36 (13 men, 23 women) 40-49: 36 (15 men, 21 women) 50-59: 43 (22 men, 21 women) 60-69: 36 (18 H et 18 F) 70-79: 42 (22 H et 20 F)	Reproducible Anatomical landmarks: yes Subject position: yes Joint/limb position: yes Stabilization: yes Pictures: no *Reference to other articles, including Andrews et al.
McKay et al^38^	2017	N and R	Model: Citec dynamometer CT 3001; CIT Technics, Groningen, Netherlands Unit: N Maximal capacity: 500 N	Contraction type: make test Mode: compression Gravity: neutralized Measures: 3 MIVC Contraction time: 3-5 seconds Rest time: 10 seconds Verbal stimuli: yes (standardized) Evaluator: 2 experienced evaluators	UE: Shoulder: RI and RE Elbow: flexion/extension LE: Hip: ABD, RI and RE Ankle: dorsiflexion/ plantarflexion	Sample: convenience Ethnicity: multiethnic (British/European, Asian, American, African, and Aboriginal/Torres strait Islander) Activity level: NS Sex: male and female Age: Between 3 and 101 years Number: 1000 (700 adults) Number per decade: 100 (20s, 30s, 40s, 50s, 60s, 70s, and ≥80)	Reproducible Anatomical landmarks: yes Subject position: yes Joint/limb position: yes Stabilization: yes Pictures: no *Reference to supplementary data of the article 1000 norms Project: protocol of a cross-sectional study cataloging human variation
de Oliveira et al^35^	2018	R	Model: Lafayette Instrument Company HHD Unit: kg Results expressed in % Maximal capacity: NS (1335 N)	Contraction type: make test Mode: compression Gravity: against gravity Measures: 3-repeated MIVC Contraction time: NS Rest time: 20 seconds Verbal stimuli: yes Evaluator: 2 trained evaluators (3 years of clinical practice; 30 hours of training procedures and devices)	LE: Hip: ABD, extension and flexion	Sample: convenience (local university setting and the community) Ethnicity: NS Activity level: active and inactive Sex: male and female Age: 18-65 years Number: 152 (79 men, 73 women) Number per decade: Young adult (18-40 years): male: 24 active, 20 inactive female: 20 active, 19 inactive Older adults (41-65 years): male: 17 active, 18 inactive female: 17 active, 17 inactive	Reproducible Anatomical landmarks: yes Subject position: yes Joint/limb position: yes Stabilization: yes Pictures: yes
Riemann et al^33^	2010	N	Model: Hand-held Baseline 250 hydraulic push-pull dynamometer Unit: kg Results expressed in % of bodyweight Maximal capacity: NS (112 N)	Contraction type: make test Mode: mompression Gravity: neutralized for 2 positions (1 against gravity for the external rotation) Measures: 1 trial of each muscle test Contraction time: build their force 2-second period and maintain MIVC for 5 seconds Time rest: NS Verbal stimuli: no Evaluator: 2 evaluators	UE: Shoulder: RI and RE	Sample: convenience Ethnicity: NS Activity level: NS Sex: male and female Age: 20-40 years Number: 181 (90 men, 91 women) Number per decade: NS	Reproducible Anatomical landmarks: yes Subject position: yes Joint/limb position: yes Stabilization: No Pictures: yes

*Abbreviations:* ABD, abduction; ADD, adduction, LE, lower extremity; MIVC, maximal isometric voluntary contraction; NA, not applicable; NS, not specified; RE, external rotation; RI, internal rotation; UE, upper extremity.

**Table 3:** Protocol variables

**Authors (year)\Variables**				**Alveranga et al****^30^**	**Al-Abdhulwahab****^36^**	**Andrews et al****^33^****(1996)**	**Bohannon****^37^****(1996)**	**Bohannon****^38^****(1997)**	**Bohannon****^34^****(1986)**	**de Oliveira et al****^32^**	**McKay et al****^35^****(2019)**	**Riemann et a1^31^ (2010)**
**HHD**		Maximal capacity (N)		1334	1959	512	650	650	265	1335	500	112
		Model		√	√	√	√	√	√	√	√	√
		Mode (compression/distraction)		C	C	C	C	C	C	C	C	C
**Units of measure**		Kg							√	√		
		Lb				√			√			
		Newton			√	√	√	√			√	
		% of bodyweight		√		√		√		√		√
		Newton-meter										
**Testing procedures**		Verbal stimulation		√						√	√	
		Gravity eliminated			√	√	√	√	√		√	
		Rest time between trials, s		15	120	≥60		≥60		20	10	
		No. of trials		5	3	2		2	1	3	3	1
		Contraction time, s		5	7R	7R	7R	7R			3-5	7R
		Strength value used for final analysis, mean or maximum		Max	Mean	Mean		First	NA			NA
		No. of evaluators		2	1	3	1	1	1	2	2	2
**Muscle groups**	**Upper limbs**	Shoulder	Flexion			√			√			
			Extension			√		√	√			
			Abduction			√	√	√	√			
			Adduction						√			
			External rotation			√		√	√		√	√
			Internal rotation			√			√		√	√
		Elbow	Flexion			√	√	√	√		√	
			Extension			√		√	√		√	
		Wrist	Flexion						√			
			Extension			√	√	√	√			
	**Lower limbs**	Hip	Flexion	√		√	√	√		√		
			Extension	√						√		
			Abduction	√		√		√		√	√	
			Adduction	√								
			External rotation	√							√	
			Internal rotation	√							√	
		Knee	Flexion			√						
			Extension		√	√	√	√				
		Ankle	Dorsiflexion			√	√	√			√	
			Plantarflexion								√	
**Positioning**		Participant position		√	√	√		√	√	√	√	√
		Rater position									√	
		Joint/limb position			√	√		√	√	√	√	√
		Anatomical landmarks		√	√	√		√	√	√	√	√
		Stabilization			√	√		√	√	√	√	
		Pictures				1			√	√		√

*Abbreviations:* Max, maximum; NA, not applicable; R, ramped.

### Normative or reference values

Different terms were used to identify the maximal muscle strength data obtained from groups of individuals presenting with similar characteristics. Two studies used the term “normative values,”^33^^,^^34^ 1 study used the term “reference values” only,^35^ and 3 studies used both terms as synonyms.^36^, ^37^, ^37^ Two studies used the terms “preliminary baseline databases” or “preliminary information” to describe the obtained strength values,^39^^,^^40^ and 1 study reported them as data.^41^ No study provided a definition of the terms “normative” and “reference” values.

### Instruments and measures

In the included studies, measures of MIMS were collected using 8 different HHD devices: Accuforce II,^c^ MicroFET 2,^d^ Chatillon CSD400C, Citec dynamometer CT 3001,^e^ Lafayette Hand-Held Baseline 250 hydraulic push-pull dynamometer,^f^ Spark Instrument and Academics, Inc,^g^ and Nicholas Hand-Held Dynamometer.^h^ Results were most frequently expressed in Newtons (55.6% of studies) or in percentage of bodyweight (55.6% of studies), whereas other studies expressed strength results in kilograms (22.2% of studies) or pounds (22.2% of studies). No study reported results in Newton-meters. The maximal capacity of the HHD used ranged from 250 to 1959 N.

### Testing procedures

Protocols varied greatly between studies. All protocols used isometric “make” tests in compression mode. For most protocols, muscle strength evaluations were performed in gravity-neutralized positions for all muscle groups tested, with the exception of 3 studies in which some or all muscle groups were tested against gravity.^33^, ^34^, ^35^ The duration of the maximal isometric voluntary contraction for each trial varied across studies from 3 to 7 seconds, whereas the resting time varied from 10 seconds to 2 minutes. The number of repeated trials per muscle group ranged between 1 and 5 maximal isometric voluntary contractions. Verbal encouragements and stimuli were given during measurements in only 3 studies.^33^^,^^35^^,^^38^ The strength measures were performed by only 1 evaluator in 4 studies,^37^^,^^39^, ^40^, ^41^ 2 evaluators in 4 studies,^33^, ^34^, ^35^^,^^35^ and 3 evaluators in 1 study.^36^ The experience of the evaluators was not specified in half of the studies and for those who reported it, experience level differed greatly (3-10y using HHD).

### Muscle groups

There is considerable variability in muscle groups tested in the 9 studies analyzed. Two studies reported strength measurements of upper limb muscle groups only,^33^^,^^40^ 3 reported for lower limbs only,^33^^,^^35^^,^^39^ and 4 studies recorded data for both upper and lower limbs.^36^, ^37^, ^38^^,^^41^ Muscle groups tested in upper limbs included flexors/extensors, abductors/adductors and internal/external rotators of the shoulder, and elbow and wrist flexors and extensors. Regarding lower limbs, tested muscle groups were the flexors/extensors, abductors/adductors and internal/external rotators of the hip, the flexors/extensors of the knee, and the dorsi/plantar flexors of the ankle. In the 9 studies included, strength data were available for both sexes in all muscle groups at least once, except for the wrist flexors, which were only available for women. Plantar flexors, shoulder and hip adductors, and wrist flexors are the muscle groups for which strength data are poorly documented.

### Participants

Convenience samples of participants were recruited for all studies included in the scoping review. In most of them, ethnicity was not specified. In the study by Al-Abdulwahab,^39^ participants were all Saudi Arabian men, wheras McKay et al^38^ included participants of diverse ancestry of whom the majority were European/British, American, Asian, African, or Aboriginal/Insular of Torres Strait. Sample size ranged from 31 to 1000 participants, and the number of participants per decade of age was highly variable. Two studies did not specify the age categories of their participants,^34^^,^^40^ and 6 separated the number of participants into decades such as 20 to 29 years, 30 to 39 years, 40 to 49 years, 50 to 59 years, 60 to 69 years, and 70 to 79 years.^33^^,^^36^, ^37^, ^38^, ^39^^,^^41^ De Oliveira et al^35^ separated the participants into 2 groups: younger (18-40y) and older adults (41-65y). In all studies, the number of participants in each decade ranged from 13 to 100. Participants included in the studies were aged between 18 and 101 years old; only 2 studies included participants aged 80 years or older.^38^^,^^39^ Regarding the sex of the participants, 3 studies were only interested in strength measurements of certain muscle groups in women,^33^^,^^40^^,^^41^ 1 reported strength values only in men,^39^ and the other studies reported reference values of muscle strength for both sexes.^34^, ^35^, ^36^, ^37^, ^38^

### Positioning and protocol reproducibility

Seven of the included studies provided sufficient information to reproduce the protocol used, particularly the position of the participant for muscle testing, the limb and joint positions during the measurement process, the anatomic landmarks used for the placement of the dynamometer, and the stabilization of the segments. Additionally, McKay et al^38^ described the evaluator's position. Only 4 studies included pictures.^34^, ^35^, ^36^^,^^37^ Four studies referred to other published article protocols by the same research group where all the information needed to reproduce the protocol is available.^37^^,^^38^^,^^40^^,^^41^ Most studies provided sufficient details to reproduce the protocol used, which allowed us to determine that there does not seem to be a consensus on standard protocols to measure maximal muscle strength. Tables 4 and 5 present the positioning for each muscle group in each study.

**Table 4:** Positioning for muscle testing (upper limb muscle groups)

			**Studies**					
**Muscle Groups**			**Andrews et al****^36^**	**Bohannon****^41^**	**Bohannon****^37^**	**Bohannon****^40^**	**McKay et al****^38^**	**Riemann et al****^34^**
**Shoulder**	**Flexion**	Gravity eliminated	√			√		
		Shoulder and elbow position	90 degrees flexed 0 degrees			90 degrees flexed		
		Participant position	S			S		
		Stabilization	Axillary region			Shoulder		
		Anatomical landmark	Proximal to epicondyle			Proximal to elbow on flexor surface of arm		
	**Extension**	Gravity eliminated	√		√	√		
		Shoulder and elbow position	90 degrees flexed Flexed		90 degrees flexed Flexed	90 degrees flexed		
		Participant position	S		S	S		
		Stabilization	Superior aspect of shoulder		Superior aspect of shoulder	Shoulder		
		Anatomical landmark	Proximal to epicondyle		Proximal to epicondyle	Proximal to elbow on extensor surface of arm		
**Shoulder**	**Abduction**	Gravity eliminated	√		√	√		
		Shoulder and elbow position	45 degrees abd 0 degrees		45 degrees abd 0 degrees	45 degrees abd 0 degrees		
		Participant position	S		S	S		
		Stabilization	Superior aspect of shoulder		Superior aspect of shoulder	Trunk		
		Anatomical landmark	Proximal to lateral epicondyle		Proximal to lateral epicondyle	Proximal to elbow; lateral surface of arm		
	**Add.**	Gravity eliminated	NA			√		
		Shoulder and elbow position				45 degrees abd 0 degrees		
		Participant position				S		
		Stabilization				Trunk		
		Anatomical landmark				Proximal to elbow; medial surface of arm		
**Shoulder**	**External rotation**	Gravity eliminated	√		√	√	√	√ or not
		Shoulder and Elbow position Forearm position	45 degrees abd 90 degrees flexed		45 degrees abd 90 degrees flexed	Beside trunk 90 degrees flexed Neutral supination	Neutral 90 degrees flexed	P: 90 degrees abd SU: 30 degrees abd/scaption/diag SU: 0 degrees abd 90 degrees flexed Pronated (90 degrees) or neutral
		Participant position	S		S	S	SU	P or SU
		Stabilization	Elbow		Elbow	Arm	None	Arm
		Anatomical landmark	Proximal to styloid process		Proximal to styloid process	Proximal o wrist joint on extensors surface of arm	Proximal to wrist crease, e of extensors surface	1.3 cm proximal to ulnar styloid process
	**Internal rotation**	Gravity eliminated	√			√	√	√ or not
		Shoulder and elbow position	45 degrees abd 90 degrees flexed			Beside trunk 90 degrees flexed Neutral supination	Neutral 90 degrees flexed	P: 90 degrees abd SU: 30 degrees abd/scaption/diag SU: 0 degrees abd 90 degrees flexed Pronated (90 degrees) or neutral
		Participant position	S			S	SU	P or SU
		Stabilization	Elbow			Arm	None	Arm
		Anatomical landmark	Proximal to styloid process			Proximal o wrist joint on flexor surface of arm	Proximal to wrist crease, flexors surface	1.3 cm proximal to ulnar styloid process
**Elbow**	**Flexion**	Gravity eliminated	√		√	√	√	
		Shoulder and lbow position Forearm and wrist position	Neutral 90 degrees flexed Supinated		Neutral 90 degrees flexed Supinated	Beside trunk 90 degrees flexed Neutral supination Neutral wrist	90 degrees flexed	
		Participant position	S		S	S	S	
		Stabilization	Superior aspect of shoulder or arm		Superior aspect of shoulder or arm	Arm	Subject holds bed with contralateral hands	
		Anatomical landmark	Proximal to styloid process		Proximal to styloid process	Proximal to wrist joint on radial surface of forearm	Flexor surface of forearm, proximal to wrist crease	
	**Ext**	Gravity eliminated	√		√	√	√	
		Shoulder, elbow and forearm position	Neutral 90 degrees flexed Neutral		Neutral 90 degrees flexed Neutral	Beside trunk 90 degrees flexed Neutral supination Neutral wrist	90 degrees flexed	
		Participant position	S		S	S	S	
		Stabilization	Anterior aspect of shoulder or arm		Anterior aspect of shoulder or arm	Arm	Participant holds bed with contralateral hands	
		Anatomical landmark	Proximal to lat styloid process		Proximal to lat styloid process	Proximal to wrist joint on ulnar surface of forearm	Extensor surface of forearm, proximal to wrist crease	
**Wrist**	**Flexion**	Gravity eliminated				√		
		Shoulder and elbow position Forearm and wrist position				Beside trunk 90 degrees flexed Neutral supination Neutral wrist		
		Participant position				S		
		Stabilization				Arm and forearm		
		Anatomic landmark				Proximal to MCP joints on extensor surface of hand		
	**Extension**	Gravity eliminated	√		√	√		
		Shoulder and elbow position Wrist position Fingers position	Neutral 90 degrees flexed Neutral Relaxed		Neutral 90 degrees flex Neutral	Beside trunk 90 degrees flexed Neutral supination Neutral wrist		
		Participant position	S		S	S		
		Stabilization	Distal forearm		Distal forearm	Arm and forearm		
		Anatomic landmark	Proximal to MCP joints		Proximal to MCP joints	Proximal to MCP joints on flexor surface of hand		
	

*Abbreviations:* Abd, abduction; Add, adduction; diag, diagonally; Ext, extension; lat, lateral; MCP, metacarpophalangeal; NA, not applicable; P, prone; S, supine; SU, sitting upright.

**Table 5:** Positioning for muscle testing (lower limbs muscle groups)

			**Studies**						
**Muscle Groups**			**Alveranga et al****^33^**	**Al-Abdhulwahab****^39^**	**Andrews et al****^36^**	**Bohannon****^41^**	**Bohannon****^37^**	**de Oliveira et al****^35^**	**McKay et al****^38^**
	**Flexion**	Gravity eliminated			√		√		
**Hip**									
		Hip and knee position CLL			90 degrees flex Relaxed Neutral		90 degrees flex Relaxed Neutral	Neutral Bending over its edge; CLL knee flexed, foot on the table	
		Participant position	SU		S		S	S	
		Stabilization			Pelvis		Pelvis	Hands holding table; Waist strap	
		Anatomical landmark	5 cm above upper border patella		Femoral condyles		Femoral condyles	Superior to the patella on ant thigh region	
	**Ext**	Gravity Eliminated							
		Hip and knee position CLL						Slight ER; 90 degrees flexed	
		Participant position	P					P	
		Stabilization						Waist strap	
		Anatomical landmark	5 cm above medial malleoli					Distal posterior thigh region	
**Hip**	**Abd**	Gravity eliminated			√				√
		Hip and knee position CLL			Neutral Neutral Neutral			20 degrees abd, 10 degrees ext and hip neutral rotation	10 degrees abd Knee extended
		Participant position	S		S			SL	S
		Stabilization			CLL help in neutral			Waist strap	Participant holds edge of bed with both hands
		Anatomical landmark	5 cm above proximal border lateral malleoli		Lateral femoral condyles			Superior to the lateral malleoli	5 cm proximal to the lateral malleoli
	**Add**	Gravity eliminated							
		Hip and knee position CLL							
		Participant position	S						
		Stabilization							
		Anatomical landmark	5 cm above proximal border medial malleoli						
**Hip**	**External rotation**	Gravity eliminated							√
		Hip and knee position							90 degrees flexed 90 degrees flexed
		Participant position	SU						SU
		Stabilization							Participant holds bed with hands; stabilizes knee
		Anatomical landmark	5 cm above proximal border medial malleoli						5 cm proximal to the medial malleoli
	**Internal Rotation**	Gravity eliminated							√
		Hip and knee position							90 degrees flexed 90 degrees flexed
		Participant position	SU						SU
		Stabilization							Participant holds edge of bed with both hands; stabilizes knee
		Anatomical landmark	5 cm above proximal border lateral malleoli						5 cm proximal to the lateral malleoli
**Knee**	**Flexion**	Gravity eliminated			√				√
		Hip and knee position			90 degrees flexed 90 degrees flexed				90 degrees flexed 60 degrees flexed
		Participant position			SU Hands resting in laps				SU
		Stabilization			shoulder by assistant				Participant holds edge of bed with both hands;
		Anatomical landmark			Proximal to malleoli				10 cm proximal to the heel
	**Ext**	Gravity eliminated		√	√		√		√
		Hip and knee position		90 degrees flex 90 degrees flex	90 degrees flex 90 degrees flex		90 degrees flex 90 degrees flex		90 degrees flexed 60 degrees flexed
		Participant position		SU Hands across the chest	SU Hands resting in laps		SU Hands resting in laps		SU
		Stabilization		Belt across the waist	shoulder by assistant		shoulder by assistant		Participant holds edge of bed with both hands;
		Anatomical landmark		Proximal to malleoli	Proximal to malleoli		Proximal to malleoli		5 cm proximal to the ankle joint
**Ankle**	**Dorsiflexion**	Gravity eliminated			√		√		√
		Hip, knee, and ankle position			0 degrees 0 degrees 0 degrees		0 degrees 0 degrees 0 degrees		0 degrees 0 degrees Plantar flexion mid-range
		Participant position			S; foot off table		S; foot off table		LS
		Stabilization			Knee maintained in full ext		Knee maintained in full ext		Lower limb proximal to ankle joint
		Anatomical landmark			Proximal to MTP		Proximal to MTP		Dorsal surface of the foot proximal to the MT head
	**Plantar flexion**	Gravity eliminated							√
		Hip, knee and ankle position							0 degrees 0 degrees Plantar flexion
		Participant position							LS
		Stabilization							Lower limb proximal to ankle joint
		Anatomical landmark							Plantar surface of the foot proximal to the MT head

*Abbreviations:* Abd, abduction; Add, adduction, ant, anterior; CLL, contralateral limb; ER, external rotation; Ext, extension; flex, flexion; LS, long sitting; MT, metatarse; MTP, metatarsophalangeal; P, prone; S, supine; SL, side lying; SU, sitting upright.

## Discussion

The aim of this scoping review was to identify and map the existing body of literature regarding MIMS reference values of upper and lower limb muscle groups obtained with HHD in healthy adults. Only 9 studies met the inclusion criteria and were included in the scoping review and further analysis. In light of the results of these studies, certain MIMS reference values were established in healthy men and women between the ages of 18 and 101 years old using a HHD protocol for a variety of muscle groups of the upper and lower limbs. Unfortunately, these studies present several shortcomings that significantly restrict their use as valid reference values.

The first research question of this study was to identify whether consensus or consistency exists in the use of the terms “reference value” vs “normative value.” This scoping review suggests that there is indeed no consensus in this regard in the literature. To determine if muscle strength is considered “normal” for a given individual of a given age and sex, the measured value must be compared with a value considered to be the norm. This reflects an unfounded assumption that there is a certain universality to the construct of muscular strength. In addition, it is to be noted that the terms “reference values” and “normative values,” which are often used as synonyms in the literature, are 2 distinct concepts that are worthy of discussion. Normative values are defined as values “of, relating to, or determining norms or standards,” which in turn are defined as “a set standard of development or achievement usually derived from the average or median achievement of a large group.”^42^ Such values should be obtained from a very large cohort. Most of the studies included in this scoping review involved specific and fairly homogeneous samples of the population, with distinct characteristics. The term “reference values” is defined as the values obtained from individuals presenting conditions that are similar to that of the tested subject and well described, in circumstances that are well controlled, thus allowing adequate comparison and interpretation of the values obtained from the test.^43^ It may therefore be more appropriate to identify the values obtained from MIMS testing as reference values to be used for comparisons with individuals showing similar characteristics.

Regarding the second research question of this study, although one would expect muscle strength in adults to be well documented, this does not appear to be the case in manual dynamometry; there are many gaps in the studies published on the subject. Several limitations are related to the type of devices used to collect strength measurements as well as the procedures surrounding their use. As mentioned above, the type of device used was highly diverse. Eight different HHD devices were used in the included studies, all with different characteristics (units of measurement, upper force limit, device design [attachments, handles], compression, or traction mode), restricting comparison of the values obtained with each. Consequently, it is impossible to claim that the reference values with one device or another would be equivalent without knowledge of the concomitant validity between tools. This severely limits the clinical use of the existing reference values presented in these studies. The upper force measuring limit of the devices, also highly variable (250-1959 N), compromises the accuracy of measures in muscle groups with capacity that exceeds the measurement ceiling, as is the case for the knee extensors. Some studies included participants who generated forces above the dynamometer's upper limit of measurement, creating a ceiling effect that invalidates the mean values obtained for the muscle group involved.^36^^,^^37^^,^^40^ Therefore, these values cannot be taken into consideration for comparison.

Another major limitation in the current literature on HHD strength values is that these values are reported in units of force (kg or N) rather than torque (Newton-meters), making it impossible to use these values for comparison purposes, which is the main reason for establishing reference values. Indeed, no included study considered the anthropometric characteristics of the participants, which have an important influence on the torque that could be generated. The length of the lever arm (ie, the perpendicular distance between the placement of the HHD and the axis of rotation of the tested segment) is an important parameter as it takes individual differences in body segment length into account in the determination of the tensile force generated. For example, Alvarenga et al^33^ showed stronger hip flexors than hip extensors, which is unlikely considering that when controlling for lever arm and muscle length, the hip extensors are almost twice as strong as the hip flexors in isometric or in low velocity testing conditions.^20^^,^^23^^,^^24^ This observed difference could be explained by the more proximal placement of the dynamometer for the hip flexors than the hip extensors, resulting in a shorter lever arm for the flexors and therefore a greater force measurement in Newtons on the dynamometer. Had torque been calculated, results could have been quite different. This example demonstrates the importance of measuring the lever arm and of expressing results in torque rather than in units of force. Also, and surprisingly, some studies report strength data as a percentage of body weight. The rationale for doing so is not explained, and the clinical meaning of using such a ratio or percentage should be clearly described to make this percentage a significant biomarker of muscle impairments.

From this scoping review, it appears that reference values are not available for both sexes for muscle groups such as the radial and ulnar deviators of the wrist, the ankle evertors/invertors, and the flexors/extensors and abductors/adductors of the fingers. This highlights the lack of muscle strength reference values for distal muscle groups in the literature. In addition, no MIMS reference values were found for the wrist flexors in men. Although these muscle groups are less often evaluated in clinical settings, they can be a good indicator of weakness and diagnostic criteria for several neuromuscular diseases or musculoskeletal disorders. This supports the importance of paying closer attention to these muscle groups.

One of the research questions of this scoping review was to determine whether consensus exists regarding the protocols and methodologies used for muscle testing with HHD to obtain references values. Although most of the studies provided a description of the protocols, some of the muscle testing positions present measurement biases, such as evaluation of MIMS of certain muscle groups in positions against gravity or with insufficient joint stabilization. In addition to increasing the evaluator's role in achieving stability of the participant and the presence of cocontractions, testing muscle strength against gravity leads to an underestimation of the strength values obtained. In such a case, the weight of the limb or segment evaluated should be subtracted from the force exerted to obtain a valid result, which is clinically impractical. Alvarenga et al^33^ and de Oliveira et al,^35^ who tested hip muscle groups against gravity, as well as Riemann et al,^34^ who tested the external rotators of the shoulder in prone position, did not take the weight of the segment into account. Such methods render the reference values obtained invalid for between-subject comparisons, especially for comparisons with other studies where gravity was eliminated.

Stabilization of the subject and the HHD is essential to ensuring good content validity of maximal values obtained in an isometric condition. When stabilization is insufficient, certain compensatory movements that influence the amount of force generated by the person can be observed. In addition, the balance between the force exerted by the subject and/or the rater's ability to properly resist is not respected, inducing a subtle movement of the joint and the segment. Therefore, the muscle length and consequently the strength values are modified. Some muscle groups like the knee extensors or the hip flexors, extensors, and abductors are very strong, and it is unlikely that a clinician would have the capacity to resist the force generated by these muscle groups in compression mode without any additional stabilization.^20^^,^^23^ Indeed, in some studies (eg, Al-Abdulwahab et al^39^), the evaluator used straps to stabilize the segment and minimize unwanted hip, pelvic girdle, and trunk movements during knee extension testing. For the same muscle group, Andrews et al^36^ and Bohannon^37^ had an assistant to help stabilize the trunk for the same reasons. Yet, these procedures do not increase the ability of the evaluator to resist the force exerted by the individual.^36^, ^37^, ^38^, ^39^ Only de Oliveira et al^35^ used a belt strap made from inelastic material for better positioning of the HHD and minimization of the evaluator's effort during strength measurement of the hip flexors, extensors, and abductors.^35^ However, the landmark for the positioning of the strap was not described in the paper, limiting the reproducibility of the protocol.

Other characteristics of the strength measurement protocols could also lead to measurement biases, such as the absence of verbal stimulation/motivation during the measurements, the duration of rest periods between each trial, and the contraction time. Many studies included in the review did not use verbal stimulation during the strength measurement or do not mention it; yet motivation can affect the force generated by the participant, increasing maximal strength values. Indeed, Jung et al^44^ showed that static grip strength was significantly higher with the use of verbal encouragement. Furthermore, there is no consensus among studies concerning optimal rest time between trials. De Salles et al^45^ showed that when executing repeated maximal strength assessments, 1 minute rest intervals are sufficient to then complete a second attempt of a 1 repetition maximum bench press or back squat. However, these concentric exercises require a high level of neuromuscular coordination and cannot be compared with maximal isometric contractions. No evidence has been found in the literature about repeated maximal isometric voluntary contractions. In this scoping review, some studies used an intertrial rest time of less than 1 minute; this may have affected recovery, but more research on the subject is needed.^33^^,^^35^^,^^38^

Regarding the characteristics of the participants, although the study samples included participants aged between 18 and 101 years, some studies did not report the values according to decade,^40^ and others stratified the values into large age groups.^35^ This latter approach represents a way of reporting reference values that may tend to underestimate strength values of the younger participants and overestimate the values of the older, reducing the external validity of the data collected. Some studies did not specify the activity level of the participants, which is another limitation considering that the training volume and types of activity practiced can significantly affect muscle strength capacity.

### Study limitations

The most important limitation of this study is that this scoping review focuses on muscle strength reference values obtained with HHD excluding grip strength; therefore, the results of this study cannot be generalized to reference values obtained with other types of dynamometers, including isokinetic or pinch/grip test devices. Being a scoping review, the methodological quality of the studies retained has not been assessed and the assessment of methodological limitations or risk of bias of the evidence was not performed.

## Conclusions

This scoping review, conducted with existing methodological standards for the conduct reporting, and appraisal of scoping reviews, showed that the existing literature regarding reference values of MIMS obtained with HHD in adults is scarce and that there are many gaps with respect to methodology, particularly no use of moments of force. This gap related to reporting force values instead of moment of force is a major concern, as it does not allow the force values reported in the literature to be considered valid reference values that can be used in the clinic in their current form. Future research on the establishment of a comprehensive set of HHD reference values using a well described standard protocol with known psychometric properties is needed to render the HHD a useful clinical tool.

## Suppliers

a. Medup; Atlas-Médic.b. Chatillon; Ametek.c. Accuforce; Ametek.d. MicroFET; Ametek.e. Citec dynamometer; CITEC, DS.f. Lafayette Hand-Held Baseline 250 hydraulic push-pull dynamometer; Lafayette Instrument Company.g. Spark Instrument and Academics, Inc; Spark Instruments.h. Nicholas Hand-Held Dynamometer; Lafayette Instrument Company.

## Appendix Supplementary materials

Image, application 1

Image, application 2
