# Supplementary material for: What is Known About Muscle Strength Reference Values for Adults Measured by Hand-Held Dynamometry: A Scoping Review
Source: Arch Rehabil Res Clin Transl. 2021 Dec 7;4(1):100172. doi: 10.1016/j.arrct.2021.100172 (PMC8904874; doi:10.1016/j.arrct.2021.100172)
Supplement: Supplementary file 1 [file mmc1.pdf]

Supplementary Figure 1. Concept plan

Concept 1: Normative data/reference values

**Free vocabulary** (key words in English)		**Controlled Vocabulary** (Databases thesaurus)				
		**CINAHL Descriptors**		**MeSH (MEDLINE)**		**Emtree (Embase)**
Normal<br>Normative<br>Reference<br>Value(s)<br>Range(s)	OU (OR)	Reference values	OU (OR)	Reference values	OU (OR)	Normal value

ET (AND)

Concept 2 : Isometric muscle strength

**Free vocabulary** (key words in English)		**Controlled Vocabulary** (Databases thesaurus)				
		**CINAHL Descriptors**		**MeSH (MEDLINE)**		**Emtree (Embase)**
Isometric<br>Muscle(s)<br>Strength<br>Torque<br>Moment	OU (OR)	Isometric contraction<br>Muscle strength	OU (OR)	Isometric contraction<br>Muscle strength (no explosion)	OU (OR)	Muscle isometric contraction<br>Muscle Strength

ET (AND)

Concept 3 : Hand-held dynamometry

**Free vocabulary** (key words in English)		**Controlled Vocabulary** (Databases thesaurus)				
		**CINAHL Descriptors**		**MeSH (MEDLINE)**		**Emtree (Embase)**
Handheld<br>Hand-held<br>Manual<br>Portable<br>Dynamometer<br>Dynamometry	OU (OR)	Dynamometry	OU (OR)	Muscle strength dynamometer	OU (OR)	Dynamometer (no explosion)
